# Short-term surgical outcomes of rectal adenocarcinoma surgical treatment in Latin America: a multicenter, retrospective assessment in 49 centers from 12 countries

**DOI:** 10.1007/s00384-024-04763-z

**Published:** 2024-12-23

**Authors:** LATAM collaborative colorectal surgery consortium, Avellaneda Nicolas, Sebastian Valdivieso, Fabio Leiro, Marcos Gonzalez, Marcelo Viola Malet, Helio Moreira Junior, Reinaldo Isaacs, Eddy P. Licango-Naranjo, Augusto Carrie, Gianluca Pellino, Spinelli Antonino, Antonio Caycedo-Marulanda, Mateo Santillán, Delfina Berasategui, Camila Bustamante Mayne, Martina Brun, Diana Alejandra Pantoja Pachajoa, Matías Parodi, Rosario Martiarena, Sergio Schlain, Camila Bras Harriott, Nicolas Rotholtz, Maximiliano Bun, Analía Inés Potolicchio, Yenny Quiroga, Federico Héctor Enrique Carballo, Pablo Farina, Carlos Vaccaro, Marcos González, Simón Sedziszow, Gaston Ortigueira, Tomas Seip, Oscar Dalzio Brosutti, María Soledad Cian, Fabio Oscar Leiro, Romina Bianchi, Julieta Yanet Espino Campagna, Mariela Cedermas, Pablo Arbios, Agustín Alesandrini, Juan Alberto Perriello, Javier Ignacio Villaggi, Florencia Barbero, Juan Manuel Giordanino, Gustavo Adrián Nari, Juan Pablo Muñoz, Natalia Mira Gesto, Federico Medina, Javier Santiago Minoldo, Gerardo Zanoni, Cristian Nicolas Lucas, Debra Nielsen, Juan Manuel Sotelo, Ana Inés Leone, Federico Posner, Diego Hernán Barletta, Christian Abel Ferrufino Méndez, Rogerio Serafim Parra, Fabio Lopes de Queiroz, Daniel Mauricio Londoño Estrada, Romulo Medeiros de Almeida, João Batista de Sousa, Bruno Augusto Alves Martins, Marcela Maria Silvino Craveiro, Rogerio Saad-Hossne, Augusto Antonio Barrera Zamorano, Alejandro Nicolas Barrera Escobar, Claudio Patricio Benavides Yáñez, Benedicto Misael Ocares Urzua, Francisco Xavier Báez Rojas, Tomas Sebastian Contreras Rivas, Felipe Bellolio Roth, Daniel Moreno Miranda, Christophe Riquoir Altamirano, Andrés Iglesias Bettini, Nelson Niño Puentes, María Sofia Labrador Morales, Juan Camilo Correa Cote, Gustavo Adolfo López, Daniela Sierra Castaño, Mauricio Gonzales Dorado, Andrés Ramiro Lanza Diaz, Víctor Hugo Bruno Cao, Jessica Capre Pereira, Rosana María Babilonia Yepes, Cristina Judith Padilla Herrera, Luis Eduardo Martínez López, Maikel Adolfo Pacheco Trujillo, Javier Ernesto Barreras González, Jorge Gerardo Pereira Fraga, Rafael Torres Peña, Miguel Ángel Martínez Alfonso, Silva Segovia David Renato, Francisco David Rivadeneira Proaño, Javier López-Gómez, Aldo Hernán Jaramillo Romero, Mario Alberto López Ramírez, Pablo Zeron Pontones, Ismael Brito Toledo, Michel Hernández Valadez, Adan Ramirez-Gaona, Josue Palacio Magaña, Roberto Ángel Núñez González, Marisol Solórzano Vanegas, María Alejandra Salazar Álvarez, Carlos Alberto Medina Diarte, Ruben Dominguez Azuaga, María Alejandra Pequeño Martínez, Marcelo Laurini Zanola, Alejandro Soumastre, Federico Duran, Alexandra Duffau Furini, Felix Luis Edgar Vásquez Chirinos, Sergio Antonio Martínez-Millán, Edibel Sabrina Vicent Vásquez, Maryeli Josefina Solorzano Vásquez, Marcelo Viola Malet

**Affiliations:** 1 Montevideo, Uruguay; 2 General Surgery Department, CEMIC University Hospital, Buenos Aires, Argentina

**Keywords:** Rectal, Cancer, Neoadjuvant, Latin America, Multidisciplinary

## Abstract

**Introduction:**

Rectal cancer is a prevalent disease that requires multidisciplinary management. Results of treatment of patients suffering from this malignancy in Latin America have been scarcely reported before.

**Methods:**

A retrospective, multicenter study was conducted to report preoperative and operative characteristics of patients intervened for rectal cancer in centers from Latin America during 2015–2022, and the short-term results of treatment were analyzed. The study was open to any center receiving rectal cancer patients, irrespective of volume. The main study outcome was 30-day postoperative complications including any deviation from the normal postoperative course (Clavien Dindo I to V).

**Results:**

A total of 2044 patients from 49 centers in 12 Latin American countries were included, with a mean age of 63 years. Twenty-five percent of patients were operated in low-volume centers. Twenty-nine percent of patients had a tumor located in the low rectum, and only 53% of patients had preoperative MRI for local staging. A total of 1052 patients (52%) received neoadjuvant therapy before surgery. Eighty-six percent of patients were operated by a specialized colorectal surgeon, and 31% of patients were intervened using a conventional approach. A total of 29.9% of patients presented a postoperative complication. The anastomotic leak rate was 8.9%. Fifty-eight percent of pathology reports had less than 12 lymph nodes harvested, and 22.9% of reports did not include mesorectal quality. In the multivariate analysis, neoadjuvant therapy (OR: 1.44, *p*-value: 0.023), urgent procedures (OR: 3.73, *p*-value: 0.049), intraoperative complications (OR: 2.21, *p*-value: 0.046), advanced tumors (OR: 1.39, *p*-value: 0.036), and prolonged surgery (OR: 1.74, *p*-value: 0.004) were found to be independently related to suffering postoperative complications.

**Conclusions:**

This study includes information about the approach and results of rectal cancer management in Latin America at a large scale. In the future, this information can be used as a bridge to identify areas of improvement among rectal cancer patients’ treatment in the region.

**Supplementary Information:**

The online version contains supplementary material available at 10.1007/s00384-024-04763-z.

## Introduction

Rectal cancer is a significant public health concern worldwide, with an estimated 1.8 million new cases and 862,000 deaths each year [1]. The surgical management of rectal cancer has evolved significantly over the past decades, with improvements in surgical techniques, radiation therapy, systemic chemotherapy, and immunotherapy [2]. Despite these advances, the outcomes of rectal cancer surgery vary significantly across different regions of the world, with worse outcomes both in the short and long-term (oncologic) reported in low and middle-income countries (LMICs) [3].

Latin America (LATAM) is a diverse region with 33 countries and a population of over 600 million people. Despite the high frequency of rectal cancer in the region, with a reported incidence in 2020 of 39,917 new patients [1], little is known regarding the surgical outcomes associated with this illness. This information gap is a substantial barrier to improving the quality of care for patients with rectal cancer in the region [4].

The results of a previous study in which the same Consortium presenting this study invited surgeons from LATAM to participate in a survey to better understand the patterns of care for rectal cancer patients in the region have been published [4], which showed some worrisome results including a significant number of patients being treated in low volume centers, without access to MDT, minimally invasive surgery, etc. Furthermore, a significant number of surgeons from low-volume centers referred to use a transanal approach for rectal tumors, and almost 30% of these centers did not have access to MRI for local staging.

Obtaining reliable data on the surgical outcomes of rectal cancer patients in LMICs is challenging. The healthcare systems of these countries are frequently underfunded and understaffed, and they lack the infrastructure necessary to collect and evaluate data. In addition, there is a broad and continuously expanding range of practices for the treatment of rectal cancer, resulting in substantial variations in surgical technique and oncological therapy. Numerous cultural and socioeconomic variables may also influence healthcare access and treatment adherence, making data acquisition and analysis even more complicated [3].

We conducted a retrospective analysis of patients operated on for rectal cancer, to assess short-term outcomes of the procedures and also perioperative treatment patterns (including methods used for local and distant staging and indications for neoadjuvant therapy).

## Methods

### Ethical considerations

Institutional review boards (IRB) at each participating institution were responsible for evaluating and approving participation in the study. This is an observational, retrospective study, with no intervention in patients; therefore, specific informed consent from patients was not necessary.

### Study design and setting

General and subspeciality-trained rectal cancer surgeons across the region were encouraged through local scientific colorectal associations and the Latam Colorectal Consortium Network [5] to contribute patients’ information to a consolidated database. Participating centers were allowed to contribute data during August to December of 2022. The definition used for subspecialty-trained surgeons was given to all surgeons who received, after general surgery training, at least 2 years of additional formal training in colorectal surgery or surgical oncology.

The individuals who participated in the survey, as well as those who had participated in previous initiatives started by the Consortium [6], were then invited to participate in this study. The study was also promoted via social media using the Consortium’s Twitter account, @latamcolab, to maximize the study’s potential reach across Latin American countries. In order to avoid bias against low-volume centers, no minimum number of patients was established to qualify for study participation.

A study protocol and an electronic database were created to collect information (see “Data Collection and Management” section).

Ten specialists in the treatment of rectal cancer, including surgeons, medical and radiation oncologists, and diagnostic imaging professionals, participated in a two-round Delphi methodology to determine the database variables. The group utilized the Core Outcomes for Colorectal Cancer Surgery as established by McNair et al. through a robust consensus methodology, which incorporated all the necessary data in the study [7]. Rectal cancer or adenoma was defined as any new neoplastic lesion histologically confirmed to be of adenomatous origin and within 15 cm from the anal verge.

### Participating site profiles

Supplementary Table 1 summarizes the facilities for each participating center, including the number of beds, access to multidisciplinary teams (MDT) for discussion of rectal cancer patients, and other relevant points. This information was included in a separate form and was mandatory for each center to complete in order to be considered eligible to participate in the study.

Once the database and protocol were created, a second audit was performed by three external independent validators to assess the completeness of the data. These external validators were world-renown, academic experts who practice Colorectal Surgery in high-volume centers outside Latin America (US and Europe).

This manuscript was written following the STROBE guidelines for reporting observational studies.

### Eligibility criteria

Patients who had undergone rectal resection for a biopsy-proven rectal adenoma or adenocarcinoma between January 1, 2015, and December 31, 2022, and were at least 18 years old were eligible for the study. This included patients with locally advanced and early rectal cancer who underwent a total mesorectal excision (TME) requiring any combination of neo and adjuvant therapy, as well as those who underwent a local resection of the rectum (either conventional transanal or transanal endoscopic surgery [TES] regardless of the platform selection, rigid or flexible). Patients undergoing elective or urgent surgical procedures qualified. In addition, patients who underwent one additional procedure during the same surgical event (such as hepatic metastasectomy) were included.

Exclusion criteria were (1) patients that underwent surgery for tumors other than rectal adenoma or adenocarcinoma, (2) squamous cell carcinoma of the anus, (3) patients who received a palliative procedure only (and no rectal resection), (4) patients that required multi-visceral resection (more than one resection apart from the rectum), (5) patients receiving pelvic exenterations (anterior or posterior), and (5) patients who underwent surgery at a non-participating center.

**Fig. 1:**
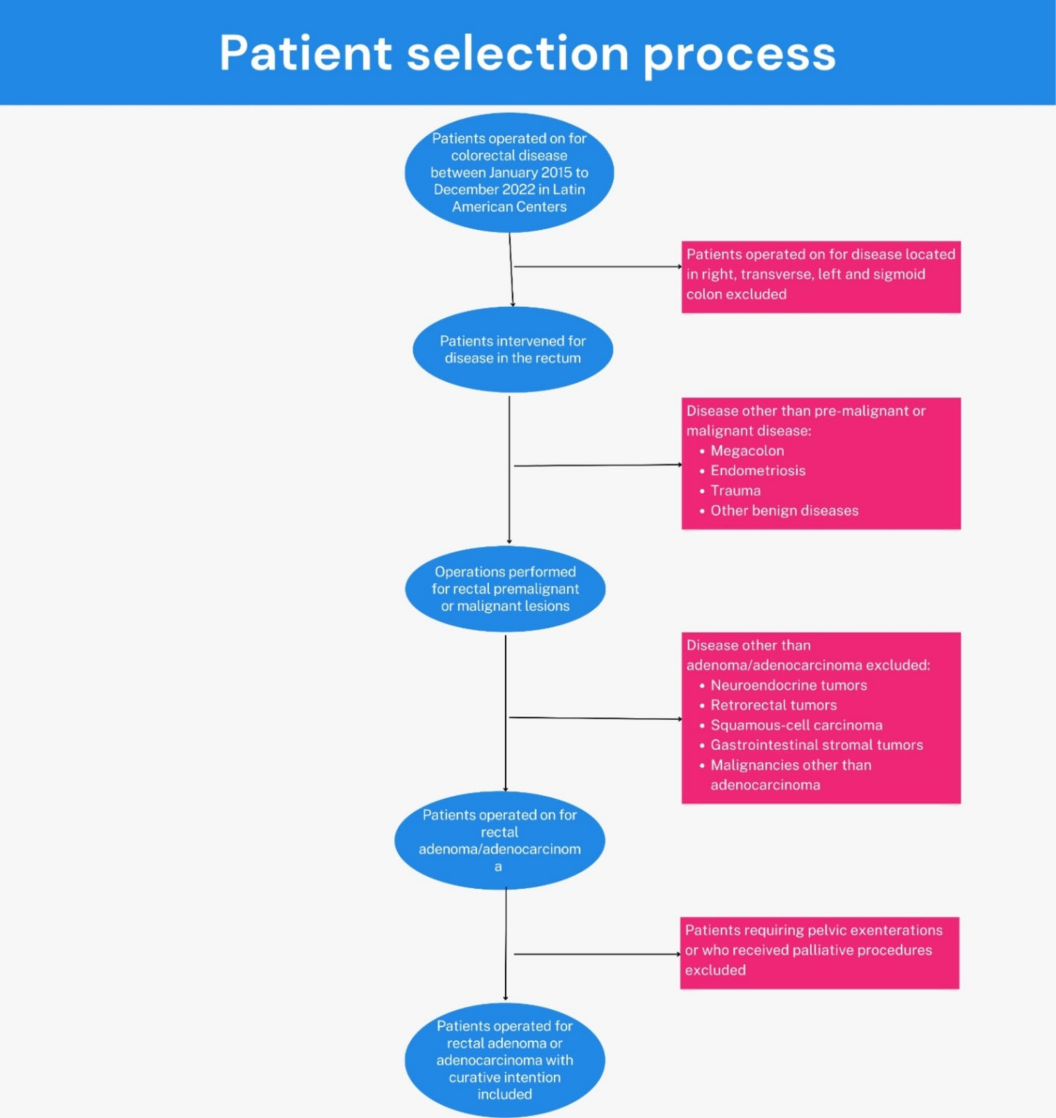
Flowchart showing the patient selection process

Figure 1 Explains the patient selection process.

### Data collection and management

Patient demographics, clinical characteristics, perioperative results, operative procedures, and short and long-term outcomes were collected in an electronic database. (RedCap, Research Electronic Data Capture, Vanderbilt University ®) designed for this purpose.

Each center designated a principal investigator who was responsible for auditing the integrity of data entered into the database. In addition, each center could recruit additional investigators to assist with data input.

### Variables analyzed

- Preoperative variables included (1) demographics (age, gender, body mass index [BMI], smoking—defined as patients consuming at least 1 cigarette per day at the time of surgery, comorbidities stratified by the World Health Organization performance status and Charlson comorbidity score); (2) previous abdominal procedures; (3) American Society of Anesthesiology score (ASA); (4) requirement of emergent surgery; (5) operator’s level of specialization (general surgeon or colorectal surgeon); (6) volume of surgeries performed annually, dividing centers in high and low volume using a cut-off value of 10 cases per year as suggested by Link et al. [8]; (7) mechanical bowel preparation and oral antibiotics prior to surgery. Patients with anemia at the time of surgery were also identified, which was defined by each hospital based on its country’s parameters. Albumin levels measured in gr/dl were also measured, considering an albumin less than 3 g/dl as low.
- Tumor-related included information on (1) serum tumor markers (carcinoembryonic antigen—CEA -, carbohydrate antigen 19–9—CA 19–9 -); (2) tumor localization; (3) distance from anal verge; (4) method used for local staging, (5) discussion in MDT prior to surgery, (6) exposure to neoadjuvant therapy and timing from neoadjuvant therapy finishing and surgery.
- Intraoperative variables included (1) procedure data (operating time, surgical approach, and conversion rates for those patients subjected to a minimally invasive approach, additional procedures performed); (2) ligation of inferior mesenteric vein (low or high as defined by Lowry et al. [9]; (3) intraoperative complications and stratification according to the CLASSIC classification [10]; (4) anastomotic characteristics (rates of primary anastomosis and diverting stomas). The decision to perform a primary anastomosis or a stoma for anastomotic protection was made by each surgeon, based on their own criteria and surgical experience. Further, mobilization of the splenic flexure was also assessed.
- Postoperative variables included (1) hospital length of stay and prolonged stay (more than 3 days) in ICU; (2) rates of postoperative complications and stratification according to Dindo-Clavien classification [11]; (3) anastomotic leak defined as per International Study Group of Rectal Cancer: Defect of the intestinal wall at the anastomotic site (including suture and staple lines of neorectal reservoirs) leading to a communication between the intra and extraluminal compartments, and further divided in minor and major leak, based on if the patient required or not a reoperation to treat the leak [12]; (4) reoperation and readmission rates (to the same hospital where the patient was operated); (5) 30-day mortality (registered in patient’s clinical chart).

Lastly, information about the specimen’s pathology report, including tumor staging and stratification into early and advanced tumors (according to AJCC classification [13]: Early tumors being stage 0-II, advanced tumors being stage III-IV). The median of lymph nodes harvested was also informed, excluding local resections.

### Outcomes

The main outcome was 30-day overall postoperative complications.

Secondary outcomes included rates of minimally invasive surgery, conversion to open surgery, intraoperative complications, hospitalization, reoperation, rehospitalization, and mortality.

### Statistical analysis

Statistical analysis was performed using Stata Software (v11.1, Statacorp, College Station, TX, USA). Categorical variables were described as percentages, whereas continuous variables were described as mean or median and range. The normality of each numerical variable was evaluated visually and with the Kolmogorov–Smirnov test.

Missing data were analyzed for pattern distribution and imputed using a regression-based multiple imputation model.

A regression analysis was performed to ascertain the effect of independent variables on 30-day postoperative complications. The odds ratio (OR) and associated 95% confidence intervals (95% CI) were reported. Univariable regression analysis was performed over variables considered clinically significant, and this analysis was used to identify the variables to include in the multivariable model: to avoid any overfitting effect, variables reaching a *p*-value of 0.01 or less were included in the multivariable regression. A *p*-value < 0.05 was considered statistically significant.

## Results

### Patient demographics and preoperative information

**Fig. 2:**
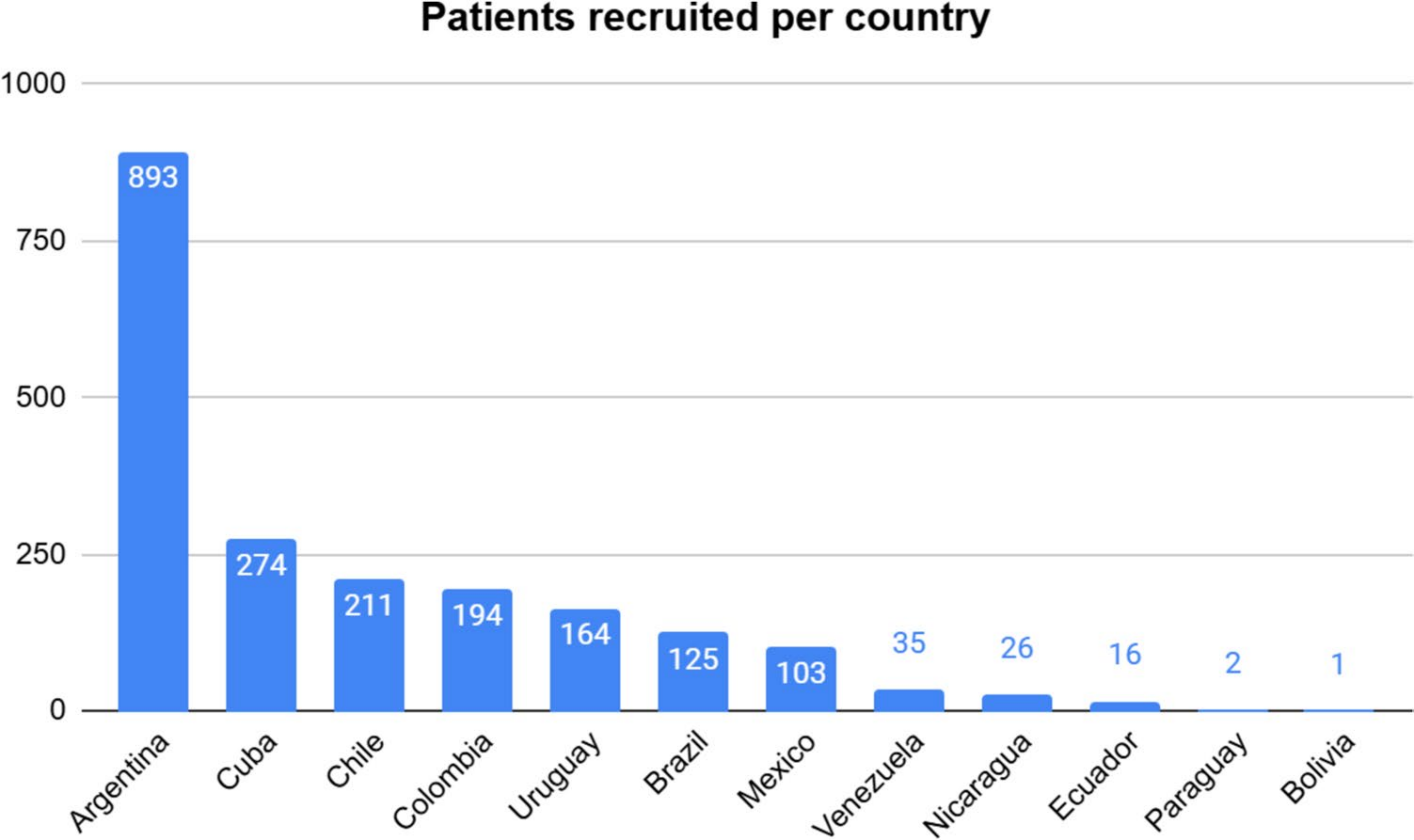
Patients included per center and country. Each column represents one specific country from Latin America. The number above the columns defines the total number of patients included by each country

A total of 2044 patients met the inclusion criteria and were included in the analysis, recruited by 49 centers in 11 Latin American countries. Figure 2 shows the number of patients recruited per country.

The mean age was 63 years (18–99), of which 912 (44.68%) were female. Five hundred two patients (24.58%) were operated in low-volume centers.

The mean BMI of patients was 26 (14.4–46.4), and 317 (19.03%) patients had a BMI higher than 30. Further, 547 patients (26.74%) had a Charlson Comorbidity Score higher than 3.

A total of 657 patients (32.51%) had anemia at the time of surgery, and only 80 patients (3.93%) were operated on in an urgent setting.

Lastly, 1703 (84.10%) of the patients received mechanical bowel preparation, whereas only 594 (29.39%) received oral antibiotics prior to surgery.

### Tumor-related variables

A total of 589 patients (29.20%) had a tumor located in the lower rectum, and the mean tumor distance from the anal verge was 8.32 cm (0–15). A total of 467 (31.96%) and 117 (16.09%) patients had increased carcinoembryonic antigen and cancer antigen 19–9, respectively.

Only 1078 patients (53.08) received an MRI for local staging of the rectal tumor, and 1557 patients (76.51%) were discussed in MDT after diagnosis and prior to treatment.

A total of 1052 patients (51.85%) received neoadjuvant therapy prior to surgery, and 94% of those completed the treatment.

Table 1 resumes the preoperative information of patients.

**Table 1:** Preoperative information

Variables	All patients *N* = 2044 (100%)	Missing values
Sex, female (*n*,%)	912 (44.68)	1
Age (mean, range)	62.74 (18–99)	17
Smoking	511 (25.09)	7
Center volume		2
Low-volume	502 (24.58)	
High-volume	1540 (75.42)	
BMI (median, range)	26.08 (14.4–46.4)	378
Low BMI (< 20)	92 (5.52)	
High BMI (> 30)	317 (19.03)	
WHO performance status > 1	810 (39.88)	13
Charlson comorbidity score		0
0–1	628 (30.69)	
2–3	871 (42.57)	
> 3	547 (26.74)	
Anemia	657 (32.51)	23
Albumin (mean, range)	3.87 (1.7–5.2)	1017
Low albumin	82 (7.98)	
Previous abdominal procedure	810 (39.80)	9
Character of surgery		10
Elective	1954 (96.07)	
Urgent	80 (3.93)	
Increased serum tumoral makers		
CEA	467 (31.96)	583
CA 19–9	117 (16.09)	1317
Tumor localization		27
Low rectum	589 (29.20)	
Midd rectum	788 (39.07)	
High rectum	640 (31.73)	
Distance from anal verge (cm, median, range)	8.32 (0–15)	261
Method used to measure distance from anal verge		254
MRI	394 (22.01)	
Colonoscopy	743 (41.51)	
Rigid proctoscopy	653 (36.48)	
Method used for local staging		13
MRI	1078 (53.08)	
Other (US, CT, PET Scan)	953 (46.92)	
Patient discussed in MDT	1557 (76.51)	9
Neoadjuvant therapy	1052 (51.85)	15
Completed neoadjuvant therapy	991 (94.38)	1
ASA classification		82
I	137 (6.98)	
II	1216 (61.98)	
III	587 (29.92)	
IV	22 (1.12)	
Bowel preparation prior to surgery		
Mechanical bowel preparation	1703 (84.10)	19
Oral antibiotics	594 (29.39)	23

### Operative information

Regarding the type of procedures performed, 1600 patients (78.74%) received an anterior resection, whereas 254 (12.50%) received an abdominoperineal excision. Lastly, 178 patients (8.76%) received a local excision.

Of those who underwent a local excision, 40% had received prior neoadjuvant therapy. Furthermore, only 24% of the patients had a T1 stage in the final pathology report, whereas 34% had a tumor stratified as pT2, and 32.09% had a pT stage higher than 2.

A total of 1748 patients (86%) were operated by specialized colorectal surgeons, and the mean time of surgical procedures was 213 min (20–587). A total of 438 patients had a prolonged operation (more than 270 min).

A total of 1405 procedures were performed using a minimally invasive approach (MI) (either laparoscopic or robotic), with a conversion rate of 8.07%, mainly due to difficulties in identifying the correct anatomical planes (51%), the presence of adhesions from prior surgeries (12%), and invasion of other organs (10%).

Intraoperative complications were identified in 3.54% of the procedures. The most frequent intraoperative complications were bleeding (15 patients), anastomotic failure (11 patients), which required a redo of anastomosis in all of them, and ureteral lesion (10 patients).

A total of 1433 patients (76.92%) had a primary anastomosis, and of these, 52% received a diverting ileostomy or colostomy. Of those patients who received a protective stoma, 21% had an upper rectal tumor. Sixty-five percent of the patients who received an anastomosis had a splenic flexure mobilization prior to it. Lastly, 167 patients in the anterior resection group did not receive a primary anastomosis.

Table 2 summarizes the operative data.

**Table 2:** Intraoperative information

Variables	All patients *N* = 2044 (100%)	Missing values
Type of surgery		12
Anterior resection	1600 (78.74)	
Abdominoperineal resection	254 (12.50)	
Local resection	178 (8.76)	
Specialization of lead surgeon		11
General Surgeon	285 (14.02)	
Colorectal Surgeon	1748 (85.98)	
Operating time (minutes, median, range)	213 (20–587)	62
Prolonged surgery (> 270 min)	438 (22.10)	
Operative approach		12
Conventional	627 (30.86)	
Laparoscopic	1382 (68.01)	
Robotic	23 (1.13)	
Conversion	113 (8.07)	5
Ligation of inferior mesenteric vein		182
Low ligation	233 (12.51)	
High ligation	1629 (87.49)	
Additional surgical procedure	311 (16.69)	181
Intraoperative complication	72 (3.54)	11
CLASSIC Minor	49 (68.06)	
CLASSIC Major	23 (31.94)	
Primary anastomosis	1433 (76.92)	181
Mobilization splenic flexure	919 (64.99)	19
Protective ileostomy/colostomy	748 (52.20)	0
Type of anastomosis		15
Stapled	1289 (90.90)	
Manual	129 (9.10)	

### Postoperative information

#### Main outcome

The overall complication rate was 29.85%, and almost half of the complications were major (Clavien-Dindo > II). The most frequent complications after surgery were prolonged postoperative ileus (130 patients), anastomotic leak (128 patients), wound infection (92 patients), and intra-abdominal abscess (82 patients).

#### Other postoperative outcomes

The median for the length of hospital stay (LOS) was 8 days (0–87). A total of 724 patients (35.68%) had a prolonged LOS (more than 1 week).

A total of 128 patients (8.93%) out of the 1433 patients who received a primary anastomosis suffered an anastomotic leak, with 70% requiring a reoperation due to this complication. The overall reoperation rate was 9.92%; the commonest reason for it was an anastomotic leak, followed by intestinal obstruction, fascial rupture, and bleeding.

A total of 142 patients (7.04) were required to be readmitted to the hospital within 30 days of surgery. Of those events, 60% were associated with surgical complications, whereas the rest were due to medical complications.

Thirty-day mortality rate was 1.98%.

Table 3 resumes postoperative outcomes of patients.

**Table 3:** Postoperative variables

Variables	All patients *N* = 2044 (100%)	Missing values
Hospitalization days (median, range)	8.37 (0–87)	15
Prolonged hospitalization (> 7 days)	724 (35.68)	
Prolonged postoperative stay in ICU	248 (12.22)	14
Postoperative complications	606 (29.85)	14
Minor complications (Clavien-Dindo I-II)	316 (52.15)	
Major complications (Clavien-Dindo > II)	290 (47.85)	
Anastomotic leak	128 (8.93)	0
Minor leak	39 (30.47)	
Major leak	89 (69.53)	
Surgical site infection	134 (6.56)	12
Reoperation	200 (9.92)	28
Rehospitalization	142 (7.04)	27
Mortality	40 (1.98)	26

### Pathology report

A total of 74 patients (3.67%) did not have a histological subtype informed in the pathology report, and 46 patients (2.28%) had an adenoma. In addition, mismatch repair status was only informed in 18.19% of all patients. The median number of harvested lymph nodes was 12, and 58.06% of the patients had less than 12 lymph nodes informed in the pathology report.

A total of 420 patients (22.91%) did not have the quality of the mesorectum informed. Furthermore, 68% of the cohort had a complete mesorectum as per the pathology report.

Table 4 includes the information related to pathology reports.

**Table 4:** Pathology report

Variables	All patients *N* = 2044 (100%)	Missing values
Histologic subtype		25
Adenoma	46 (2.28)	
Adenocarcinoma	1899 (94.06)	
Not informed	74 (3.67)	
Differentiation grade		46
Well-differentiated	543 (27.20)	
Moderately differentiated	1172 (58.72)	
Poorly differentiated	104 (5.21)	
Undifferentiated	7 (0.35)	
Not informed	170 (8.52)	
MMR		43
Proficient	333 (16.64)	
Deficient	31 (1.55)	
Not informed	1637 (81.81)	
Harvested lymph nodes (median, range)	12 (0–54)	41
% of patients with less than 12 lymph nodes harvested	1163 (58.06)	
Lymphatic invasion		30
No	1324 (65.74)	
Yes	493 (24.48)	
Not informed	197 (9.78)	
Vascular invasion		31
No	1343 (66.72)	
Yes	480 (23.85)	
Not informed	190 (9.44)	
Circumferential resection margin		205
Non compromised	1512 (82.22)	
Compromised	168 (9.14)	
Not informed	159 (8.65)	
Classification of mesorectum		211
Incomplete	47 (2.56)	
Partially complete	124 (6.76)	
Complete	1242 (67.76)	
Not informed	420 (22.91)	
Tumor budding		39
No	867 (43.24)	
Yes	220 (10.97)	
Not informed	918 (45.79)	
Extramural vascular invasion		40
No	1460 (72.85)	
Yes	130 (6.49)	
Not informed	414 (20.66)	
Perineural invasion		
No	1477 (73.41)	
Yes	336 (16.70)	
Not informed	199 (9.89)	
Resection margins		41
Compromised	202 (10.08)	
Not compromised	1729 (86.32)	
Not informed	72 (3.59)	
T-stage		33
pT0	175 (8.70)	
pT1	201 (10)	
pT2	501 (24.91)	
pT3	838 (41.67)	
pT4a	147 (7.31)	
pT4b	74 (3.68)	
Not informed	75 (3.73)	
N-stage		37
pN0	1235 (61.53)	
pN1	417 (20.78)	
pN2	213 (10.61)	
Not informed	142 (7.08)	

### Multivariate analysis

In the multivariate analysis, neoadjuvant therapy (OR: 1.44, *p*-value: 0.023), urgent procedures (OR: 3.73, *p*-value: 0.049), intraoperative complications (OR: 2.21, *p*-value: 0.046), advanced tumors (OR: 1.39, *p*-value: 0.036), and prolonged surgery (OR: 1.74, *p*-value: 0.004) were found to be independently related to suffering postoperative complications within 30 days from the original surgical procedure.

Table 5 presents the results of the multivariate analysis using different operative outcomes rates as dependent variables.

**Table 5:** Multivariate analysis considering main postoperative outcomes as dependent variables

Variables	OR	Standard error	*P* value	95% CI
MI approach				
Low volume center	0.29	0.04	< 0.001	0.22–0.38
Anemia	0.55	0.08	< 0.001	0.41–0.75
Neoadjuvant therapy	0.58	0.09	< 0.001	0.43–0.78
Urgent procedure	0.00	0.04	< 0.001	0.04–0.22
Additional surgical procedure	0.33	0.06	< 0.001	0.21–0.40
Advanced tumor	0.65	0.10	0.004	0.49–0.87
General surgeon	0.12	0.24	< 0.001	0.08–0.18
Conversion to open surgery				
Low volume center	1.72	0.45	0.039	1.03–2.87
Additional surgical procedure	2.05	0.60	0.015	1.15–3.65
Advanced tumor	1.81	0.45	0.016	1.12–2.94
Intraoperative complications				
Low volume center	2.11	0.62	0.012	1.18–3.76
Advanced tumor	1.95	0.64	0.039	1.03–3.70
Prolonged hospitalization (> 7 days)				
Low volume center	1.75	0.34	0.004	1.19–2.55
Anemia	1.54	0.28	0.019	1.07–2.22
Neoadjuvant therapy	1.72	0.30	0.002	1.22–2.41
Urgent procedure	4.29	2.90	0.032	1.14–16.20
Conversion to open surgery	1.98	0.52	0.010	1.17–3.33
Additional surgical procedure	1.75	0.41	0.018	1.10–2.77
Prolonged surgery	1.89	0.37	0.001	1.29–2.77
Postoperative complications				
Neoadjuvant therapy	1.44	0.23	0.023	1.05–1.97
Urgent procedure	3.73	2.50	0.049	1.00–13.87
Intraoperative complications	2.21	0.87	0.046	1.01–4.81
Advanced tumor	1.39	0.22	0.036	1.02–1.90
Prolonged surgery	1.74	0.33	0.004	1.20–2.52
Anastomotic leakage				
Neoadjuvant therapy	1.74	0.48	0.045	1.01–3.00
Urgent procedure	9.23	7.65	0.007	1.82–46.50
Reoperation				
Neoadjuvant therapy	1.95	0.51	0.010	1.17–3.22
High BMI	1.97	0.51	0.010	1.17–3.27
ASA > II	2.27	0.62	0.003	1.32–3.88
Rehospitalization				
ASA > II	1.86	0.55	0.033	1.05–3.32
Charlson score > 3	0.57	0.17	0.053	0.33–1.02

## Discussion

The present study reports short-term results of operative management of rectal cancer in Latin America from 2015 to 2022. Our study encompasses not only surgical outcomes but also pre and post-operative information; it also provides detailed information on staging and pathology.

Prospective cancer registries exist in high-income countries like the UK, Denmark, and Netherlands [14–16], and these registries have helped those countries improve the results of cancer treatment by planning campaigns based on dealing with problems identified in the registries.

On the contrary, it is unusual to have a cancer registry in low LMICS, usually due to a lack of resources, poor organization, and no governmental support. [17]. More recently, Argentina has managed to start its own prospective databases [18, 19], yet this initiative is relatively new, and its impact on cancer care is still to be defined. Most countries in Latin America remain with little or no information related to the surgical management of rectal cancer and its outcomes, thus no ground to base decisions to improve patient care.

For this reason, a growing number of surgeons practicing surgery in Latin American countries have led to the creation of a regional colorectal surgery consortium, which is rapidly mobilizing efforts and resources toward the collection of retrospective and prospective data that can ultimately generate the necessary knowledge and understanding to contribute to the standardization of the therapeutic practices [4–6, 17, 20, 21].

Rectal cancer is a complex disease, and treating this particular malignancy requires a multidisciplinary approach since it involves a decision-making process including different oncological regimens that frequently change as advances are made and surgical procedures which are also complex [22, 23]. For this reason, the authors have included information not only on the surgical results per se but also about centers’ facilities, accessibility to locoregional staging with high-resolution MRI, availability to discuss cases at a multidisciplinary tumor board, and synoptic pathology reports. Furthermore, center volume was also considered, since this has been described as an independent prognostic factor [24, 25].

About 25% of patients were operated on in low-volume centers, and this fact was independently related to several worse postoperative outcomes in the multivariate analysis (conventional surgery, conversion from MIS to open surgery, intraoperative complications, prolonged hospitalization). These results correlate with findings of other studies looking specifically into the relation between center volume and outcomes in colorectal cancer patients [26], even though defining the number of cases to be considered a low-volume center is challenging [27]. In the future, we intend to explore any possible association between this variable and long-term oncologic outcomes. It is also of significant interest that only 50% of the cohort received a preoperative MRI, which is nowadays considered the gold standard for locoregional staging in rectal cancer patients [28].

Similarly, half of the patients received neoadjuvant therapy, and this was found to be related to suffering postoperative complications in the multivariate analysis (OR: 1.44, *p*-value: 0.023). An increase in the risk of short-term postoperative complications had been found in prior studies [29], and it would be appropriate to look into differences in neoadjuvant schemes between centers (short- or long-course radiotherapy, total neoadjuvant therapy, time passed between finishing treatment and surgery, etc.). This analysis would also aid in assessing the need for standardization of treatment among the region.

As regards the operations, surprisingly, almost one-third of the patients were operated using an open approach. Even though this was not associated with worse postoperative outcomes in the multivariate analysis, it shows the potential benefit of training surgeons from the region in MIS. This could also impact the length of surgery, and a prolonged procedure was independently associated with complications in the multivariate analysis (OR: 1.74, *p*-value: 0.004). Nevertheless, and despite the proven benefits of this approach, we should clarify that the adoption of MIS in this type of disease has been deemed challenging in previous publications [30].

Fifteen percent of the procedures were performed by general surgeons, possibly due to the fact that most low-volume centers might not count on a colorectal specialist. This was associated with lower rates of MI approach.

An overall assessment of postoperative results shows that improvement should be achieved in most of them, including more than one-third of patients being hospitalized for more than 1 week, a high complication rate (29%), and an anastomotic leak rate of almost 10%. Further, the retrospective nature of the study probably implies that these numbers can be underestimated.

Lastly, it is relevant to mention that 40% of patients who received a local excision have received neoadjuvant therapy before (probably indicating the patients had a locally advanced disease), and only 24% of the patients had a T1 tumor in the pathology report. This raises the question of to whom and under what argument are surgeons in the region indicating local resections instead of more radical surgeries.

Limitations of this study include its retrospective nature and the fact that some patients from participating centers might have missed inclusion because of a lack of access to patient’s information. Further, significant heterogeneity in surgical practice between centers, and especially, between countries, can imply several differences in the approach to rectal cancer patients. There is no unified concept of surgical subspecialty in the region (some surgeons being trained in colorectal surgery and others as oncological surgeons). This fact highlights the importance of providing reliable data to create regional guidelines in the future for colorectal cancer that standardize the treatment of patients and training for surgeons. The definition chosen for height of ligation of the mesenteric vein is based on one specific study. Some of the confidence intervals reported in the multivariate analysis related to urgent procedures are large, which might be an effect of these types of procedures being infrequent in this cohort. Even though a significant number of centers participated in this initiative, they represent a small sample considering the vast population of Latin America. Furthermore, some countries in the region were not represented in the study, probably due to difficulties in reaching surgeons from those places using the @Latamcollab network and Social Media. Lastly, the study probably targeted surgeons with a specific interest in colorectal surgery. For these reasons, a significant representation bias can be associated with this study and needs to be corrected in the future.

Nevertheless, this manuscript includes a vast number of centers in different countries of Latin America providing information on rectal cancer treatment. Since Latin America comprises more than half a billion inhabitants, the information provided will serve the purpose of starting to understand how outcomes of rectal cancer treatment can be improved, which will affect a significant number of patients.

## Conclusion

The present study reports the outcomes of surgical treatment of a large number of patients in different centers of Latin America, including information on a lot of aspects related to the preoperative management of patients, as well as operative and postoperative outcomes.

In the future, this information can be used as a bridge to identify areas of improvement among rectal cancer patients’ treatment in the region.

## Supplementary Information

Below is the link to the electronic supplementary material.

Supplementary file1 (DOCX 8 KB)

## Data Availability

No datasets were generated or analysed during the current study.
