# Supplementary material for: Short-term surgical outcomes of rectal adenocarcinoma surgical treatment in Latin America: a multicenter, retrospective assessment in 49 centers from 12 countries
Source: Int J Colorectal Dis. 2024 Dec 23;39(1):210. doi: 10.1007/s00384-024-04763-z (PMC11663813; doi:10.1007/s00384-024-04763-z)
Supplement: Supplementary file 1 — Supplementary file1 (DOCX 8 KB) [file 384_2024_4763_MOESM1_ESM.docx]

**Supplementary table 1. Participating centers´ facilities.**

| **Variables** | **Number of centers** | **Missing values** |
| --- | --- | --- |
| **Type of center** |  | 3(5.66%) |
| Public | 23(43.4%) |  |
| Private | 25(47.17%) |  |
| Independent group | 2(3.77%) |  |
| **Number of beds** |  | 3(5.66%) |
| 0-100 | 8(15.09%) |  |
| 100-250 | 17(32.08%) |  |
| 250-400 | 11(20.75%) |  |
| 400-600 | 11(20.75%) |  |
| >600 | 3(5.66%) |  |
| **MDT** |  | 3(5.66%) |
| No | 4(7.55%) |  |
| Yes | 46(86.79%) |  |
| **Oncological treatment of patients (chemotherapy/radiotherapy) done in** |  | 3(5.66%) |
| Same institution | 21(39.62%) |  |
| Different institution | 12(22.64%) |  |
| Mainly same institution, some patients other institution | 17(32.08%) |  |
| **MRI available for local staging of rectal cancer** |  | 3(5.66%) |
| No | 7(13.21%) |  |
| Yes | 43(81.13%) |  |
| **Diagnosis of liver metastases of CRC done mainly with:** |  | 3(5.66%) |
| Abdominal ultrasound | 2(3.77%) |  |
| CT | 37(69.81%) |  |
| MRI | 11(20.75%) |  |
| Other | 0 |  |
| **Diagnosis of lung metastases of CRC done mainly with:** |  | 3(5.66%) |
| Chest X-Ray | 1(1.89%) |  |
| CT | 49(92.45%) |  |
| Other | 0 |  |
| **MIS platform available for surgery** |  | 3(5.66%) |
| No | 4(7.55%) |  |
| Yes | 46(86.79%) |  |
| **Colorectal specialized surgeon available** |  | 3(5.66%) |
| No | 6(11.32%) |  |
| Yes | 44(83.02%) |  |
| **Number of patients entered in the study** |  | 3(5.66%) |
| 0-14 | 11(20.75%) |  |
| 15-50 | 21(39.62%) |  |
| 51-100 | 9(16.98%) |  |
| >100 | 9(16.98%) |  |

**Abbreviations: MDT:** Multidisciplinary team for rectal cancer patients, MRI: Magnetic Resonance Image, CRC: Colorectal cancer, CT: Computed tomography, MIS: Minimally invasive surgery,
